# Nonreciprocal elasticity and the realization of static and dynamic nonreciprocity

**DOI:** 10.1038/s41598-020-77949-4

**Published:** 2020-12-10

**Authors:** Mohamed Shaat

**Affiliations:** Mechanical Engineering Department, Abu Dhabi University, P.O. BOX 1790, Al Ain, United Arab Emirates

**Keywords:** Mechanical engineering, Topological matter, Applied physics

## Abstract

The realization of the mechanical nonreciprocity requires breaking either the time-reversal symmetry or the material deformation symmetry. The time-reversal asymmetry was the commonly adopted approach to realize dynamic nonreciprocity. However, a static nonreciprocity requires—with no any other option—breaking the material deformation symmetry. By virtue of the Maxwell–Betti reciprocal theorem, the achievement of the static nonreciprocity seems to be conditional by the use of a nonlinear material. Here, we further investigate this and demonstrate a novel “nonreciprocal elasticity” concept. We investigated the conditions of the attainment of effective static nonreciprocity. We revealed that the realization of static nonreciprocity requires breaking the material deformation symmetry under the same kinematical and kinetical conditions, which can be achieved only and only if the material exhibits a nonreciprocal elasticity. By means of experimental and topological mechanics, we demonstrate that the realization of static nonreciprocity requires nonreciprocal elasticity no matter what the material is linear or nonlinear. We experimentally demonstrated linear and nonlinear metamaterials with nonreciprocal elasticities. The developed metamaterials were used to demonstrate that nonreciprocal elasticity is essential to realize static nonreciprocal-topological systems. The nonreciprocal elasticity developed here will open new venues of the design of metamaterials that can effectively break the material deformation symmetry and achieve, both, static and dynamic nonreciprocity.

## Introduction

Nonreciprocity has been introduced to various fields to give asymmetrical, nonlinear, and/or time non-revisal physical systems^1–9^. Optical nonreciprocity has been recently introduced to photonics, optical diodes, and insulators to give nonreciprocal transmissions of light fields^1–5^. In addition, nonreciprocity has been introduced to realize mechanical systems with topological characteristics, e.g., nonreciprocal waves^6^, static nonreciprocity^7,10^, and nonreciprocal edge states^8^. In many occasions, the nonreciprocity was achieved using nonlinear systems, which disobey the reciprocity laws, e.g., Lorentz reciprocity law^4^. Nonetheless, materials or structures that would achieve asymmetric couplings of certain fields would break the time-reversal symmetry and induce nonreciprocity without breaking the reciprocity laws^11^. This has been realized in optical systems where structures that achieved asymmetric couplings of the optical fields induced optical nonreciprocity without breaking the Lorentz reciprocity law^9^. This indicates that the violation of the reciprocity law of a physical system does not necessarily imply that the physical system is really nonreciprocal. Therefore, other verifications of the nonreciprocity would be required. Here, we demonstrate that the attainment of the nonreciprocity in mechanical systems is conditional by breaking either the time-reversal symmetry or the deformation symmetry under the same kinematical and kinetical conditions.

By virtue of the Maxwell–Betti reciprocal theorem, nonlinear materials could impart some nonreciprocal characteristics to mechanical systems. Therefore, nonlinear materials have been proposed to realize nonreciprocal mechanical systems^6,12–17^. However, breaking the Maxwell–Betti reciprocal law does not guarantee the realization of an effective nonreciprocal mechanical system^7,11,18^. An effective nonreciprocity requires special mechanisms or structures that can break the time-reversal symmetry and/or the material’s deformation symmetry under the same kinematical and kinetical conditions. For example, active materials, e.g., materials with gyroscopic effects, can achieve effective dynamic mechanical nonreciprocity^18–20^. Active materials can modulate their properties in space and time to break the time-reversal symmetry in response to external stimuli^18^. Metamaterials with gyrotropic properties exhibited time-reversal asymmetry and achieved nonreciprocal wave propagation and wave isolation^21–24^. Other examples of active materials that achieved optical and acoustic nonreciprocity have been proposed^18,25–27^. It is not only active materials but also passive materials that combine large nonlinearities and microstructural geometrical asymmetries would achieve effective nonrecirpcoity^7,18,28^. Passive materials are even better than active materials to provide compact designs of nonreciprocal systems^28^.

The current approaches of achieving nonreciprocity using passive materials only depended on employing large nonlinearity^18,28^. Some of these nonlinearity-based approaches utilized inhomogeneous materials with properties that spatially vary^29–31^ or materials with microstructural-nonlinear defects^29,32–39^ to achieve dynamic nonreciprocity. Other approaches, on the other hand, depended on large-deforming the material to activate some nonlinear bifurcations, and hence the realization of dynamic nonreciprocity^40,41^ and static nonreciprocity^7,42^. These studies provided insightful understanding of the realization of nonreciprocity in nonlinear-passive media. Nonetheless, these studies hide information on the real cause of the nonreciprocity, as it follows that the trigger of the nonreciprocity is not the material being nonlinear but the activation of special nonlinear fields. These studies also diminish the possibility of realizing nonreciprocity using linear-passive materials.

Here, we further investigate this and show the realization of nonreciprocity using linear-passive materials. We demonstrate that the static mechanical nonreciprocity can be achieved only and only if the material exhibits “nonreciprocal elasticity”. By definition, static nonreciprocity requires a passive material (non-active material). It is not a matter whether the passive material is linear or nonlinear, but the material should be specially designed to achieve nonreciprocal elasticity. We experimentally demonstrate an example of a metamaterial with nonreciprocal elasticity. In addition, we demonstrate that the realization of a nonreciprocal elasticity guarantees, both, effective static and dynamic nonreciprocity. By means of topological mechanics, we demonstrate that the static nonreciprocity requires nonreciprocal elasticity no matter what the material is linear or nonlinear. We demonstrate that not only nonlinear materials but also linear materials can achieve effective mechanical nonreciprocity, which can be verified by measuring the material’s asymmetric deformation under the same kinematical and kinetical conditions.

## Results and discussion

### Maxwell–Betti reciprocal theorem and mechanical nonreciprocity

In general, the asymmetric behavior and the nonreciprocal behavior of physical systems are two different concepts^11,43^. Despite the nonreciprocity would be—in many cases—associated with the system’s asymmetric behavior, physical systems with asymmetric behaviors are not necessarily nonreciprocal^11,43^. On the one hand, the verification of the asymmetric behavior of a physical system requires testing the system before and after the spatial inversion of the system^11^. On the other hand, the verification of the nonreciprocity of a physical system requires testing the system before and after both the time-reversal and the spatial inversion of the system^11^. This difference between the “asymmetry” and the “nonreciprocity” indicated that the nonreciprocity would require a mechanism of breaking the time reversal symmetry. Breaking the time reversal symmetry was the approach that has been adopted by many of the existing studies that demonstrated dynamic nonreciprocity (refer to Ref.^18^). Breaking the time reversal symmetry requires an active material with embedded mechanisms, e.g., gyroscopes, that can modulate their properties in space and time. Such an active material disobeys the reciprocal laws, e.g., Maxwell–Betti reciprocal theorem, because it breaks the time-reversal symmetry.

**Figure 1:**
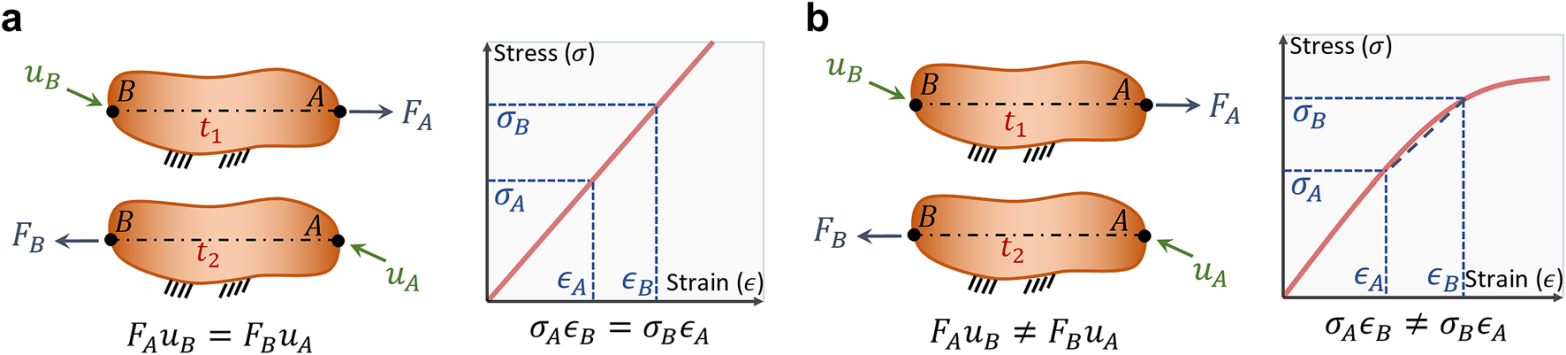
Maxwell–Betti reciprocal theorem. (**a**) Linear elastic materials versus (**b**) nonlinear elastic materials according to the Maxwell–Betti reciprocal theorem. (**a**) A linear elastic material that is subjected to two different forces, ${F}_{A}\ne {F}_{B}$. At instant ${t}_{1}$, the material exhibited a displacement at point B of ${u}_{B}$ when subjected to a force ${F}_{A}$ at point A. According to the Maxwell–Betti reciprocal theorem, we expect that the material deforms at instant ${t}_{2}$ such that its displacement at point A due to an applied force ${F}_{B}$ at point B is ${u}_{A}={F}_{A}{u}_{B}/{F}_{B}$ (thus, ${F}_{B}{u}_{A}={F}_{A}{u}_{B}$). (**b**) A nonlinear elastic material that is subjected to two different forces, ${F}_{A}\ne {F}_{B}$. This material disobeys the Maxwell–Betti reciprocal theorem such that ${F}_{B}{u}_{A}\ne {F}_{A}{u}_{B}$.

Whereas the main difference between the system asymmetry and nonreciprocity is that nonreciprocal systems break the time reversal symmetry, it is possible to achieve nonreciprocity using time-invariant physical systems, e.g., passive media. Nonreciprocity has been demonstrated using passive materials^18,28–39^, and this required, mainly, using nonlinear materials. Nonlinear materials disobey the Maxwell–Betti reciprocal theorem. The Maxwell–Betti reciprocal theorem^12^ was developed to examine the symmetry in the material’s elastic deformation under different kinematical and kinetical conditions. This theorem uses the material deformation history to expect its deformations under different loads. Thus, if the deformation of a linear elastic material at point $B$ (${u}_{B}$) due to an applied force at point $A$ (${F}_{A}$) was previously measured, we can expect its deformation at point $A$ (${u}_{A}$) due to an applied force at point $B$ (${F}_{B}$) by ${u}_{A}={F}_{A}{u}_{B}/{F}_{B}$ (Fig. 1). This indicates that the Maxwell–Betti reciprocal theorem stems from the time-reversal symmetry principle^44^. The breakdown of this theorem indicates a nonlinear material. According to the Maxwell–Betti reciprocal theorem, if the deformation of a material does not depend on its deformation history, the material is a nonlinear material, and it may break the time-reversal symmetry of physical systems.

**Figure 2:**
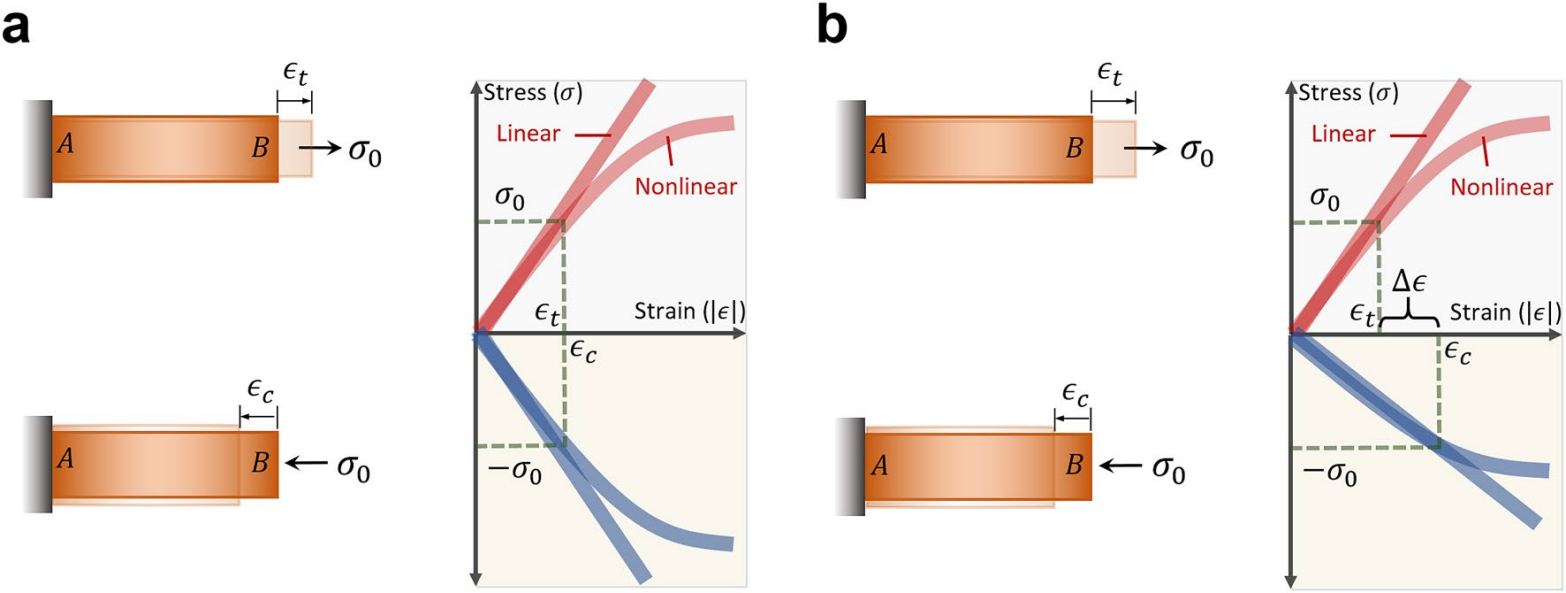
The concept of nonreciprocal elasticity. (**a**) Linear and nonlinear materials with reciprocal elasticity. The material exhibits the same elastic stress–strain curve under the tensile and compression tests. This material exhibits the same deformation ${\epsilon }_{t}={\epsilon }_{c}$ when subjected to the same axial stress ${\sigma }_{0}$. (**b**) Linear and nonlinear materials with nonreciprocal elasticity. The material exhibits two different elastic stress–strain curves under the tensile and compression tests. This material exhibits asymmetric deformation $\Delta \epsilon ={\epsilon }_{c}-{\epsilon }_{t}$ when subjected to the same axial stress ${\sigma }_{0}$.

In addition to the attainment of nonreciprocity by breaking the time reversal symmetry, nonreciprocity has been achieved by breaking the material deformation symmetry^7^. In some recent studies, the Maxwell–Betti reciprocal theorem was used to examine the breaking of the material deformation symmetry and the attainment of mechanical nonreciprocity^6,7^. By virtue of the Maxwell–Betti reciprocal theorem, the achievement of the nonreciprocity is thought to be conditional by the use of a nonlinear material. However, the breaking of the Maxwell–Betti reciprocal law does not guarantee a nonreciprocity. A nonlinear material—that disobeys the Maxwell–Betti reciprocal theorem—can achieve asymmetric deformation but under two different loading conditions. Such asymmetric deformation is weak, and it is not enough to achieve effective mechanical nonreciprocity. However, under the same loading conditions, the deformation of nonlinear materials is generally symmetric as long as the material is reciprocal (see Fig. 2a).

It follows from the previous discussion that the attainment of the nonreciprocity in mechanical systems requires breaking either the time-reversal or the deformation symmetry. The achievement of mechanical nonreciprocity using approaches of breaking the material deformation symmetry is scarce in the literature. One main reason is that the asymmetric deformation does not guarantee the attainment of nonreciprocity (i.e., the asymmetric material behavior does not mean the material is nonreciprocal). Here, we further investigate this and demonstrate that the effective mechanical nonreciprocity requires breaking the deformation symmetry under the same kinematical and kinetical conditions. The verification of the material asymmetric deformation can be done by testing the material under two different kinematical and/or kinetical conditions; nonlinear materials, in general, exhibit asymmetric deformations when tested under two different loading conditions. However, the verification of the attainment of nonreciprocity that depend on breaking the material deformation symmetry requires testing the material under the same, but spatially inverted, kinematical and kinetical conditions.

### Nonreciprocal elasticity

Here, we demonstrate that the achievement of the nonreciprocity in time-invariant mechanical systems requires a passive material with ‘nonreciprocal elasticity’. The material nonlinearity does not guarantee an effective mechanical nonreciprocity. However, a material with nonreciprocal elasticity achieves mechanical nonreciprocity no matter what the material is linear or nonlinear. We demonstrate that not only nonlinear materials but also linear materials can achieve effective mechanical nonreciprocity as long as it exhibits nonreciprocal elasticity. The verification of the nonreciprocal elasticity of time-invariant materials (passive materials) requires examining the material’s ability to break the material deformation symmetry under the same kinematical and kinetical conditions. This can be done by testing the material before and after the spatial inversion of the applied load, e.g., axial tension and compression.

Consider an elastic material that is setup under axial tensile and compression tests such that it is fixed from its side A and loaded from its side B (Fig. 2). This material would give two identical elastic stress–strain curves when it is tested under tension and compression, as shown in Fig. 2a. A material with this behavior is a “*reciprocal elastic*” material. Reciprocal elasticity is a common property of most of the natural materials including both linear and nonlinear materials. When a reciprocal elastic material is stretched or compressed by the same force, it shows the same stiffness and the same deformation (see Fig. 2a). No matter what the material is linear or nonlinear, such a reciprocal elastic material exhibits a symmetric deformation under the same applied stress ${\sigma }_{0}$ (Fig. 2a).

In Fig. 2b, we show examples of linear and nonlinear materials with “*nonreciprocal elasticity*”. Nonreciprocal elasticity can be defined as the contrast in the material’s elastic properties along two opposite directions. In other words, a nonreciprocal elastic material is the one that exhibits asymmetric elastic properties, which are different when measured along two opposite directions. A nonreciprocal elastic material modulates its stiffness depending on the direction of the applied load. Even if it is loaded with the same stress, its tensile stiffness is different than its compressive stiffness. Thus, it gives asymmetric deformation under the same loading conditions.

### Static and dynamic nonreciprocity using linear-passive materials

It was thought that the achievement of nonreciprocity using passive materials is only associated with the material nonlinearity^18,28^. However, per our previous discussion, the attainment of static nonreciprocity requires—with no any other option—a passive material with nonreciprocal elasticity, no matter what the material is linear or nonlinear. To further demonstrate this, the static nonreciprocity (${{\varvec{\upchi}}}_{s}$) and the dynamic nonreciprocity (${{\varvec{\upxi}}}_{d}$) of passive materials in terms of the nonreciprocal elasticity (${\varvec{\epsilon}}$) are determined. The equation of motion of an elastic passive material under a body force can be considered, as follows:

1 $$\nabla \cdot \left(\mathbf{C}:\mathbf{e}\right)+\rho \lambda \mathbf{u}=-\mathbf{f}$$

where $\mathbf{C}$ is the 4th-order material’s elasticity tensor, $\mathbf{e}$ is the infinitesimal strain tensor, $\mathbf{f}$ is an applied body force, and $\rho$ is the material’s mass density. $\mathbf{u}$ is the displacement field of a point belongs to the material’s domain, and $\lambda$ is the eigenvalue of the vibration of this material point about is equilibrium position. Now, let us assume that the material is subjected to two opposite (i.e., spatially inverted) forces ${\mathbf{f}}^{\left(1\right)}$ and ${\mathbf{f}}^{\left(2\right)}$ such that:

2 $$\nabla \cdot \left(\frac{1}{2}{\mathbf{C}}^{\left(1\right)}:\left(\nabla {\mathbf{u}}^{\left(1\right)}+{\mathbf{u}}^{\left(1\right)}\nabla \right)\right)+\rho {\lambda }^{\left(1\right)}{\mathbf{u}}^{\left(1\right)}-\nabla \cdot \left(\frac{1}{2}{\mathbf{C}}^{\left(2\right)}:\left(\nabla {\mathbf{u}}^{\left(2\right)}+{\mathbf{u}}^{\left(2\right)}\nabla \right)\right)-\rho {\lambda }^{\left(2\right)}{\mathbf{u}}^{\left(2\right)}=-{\mathbf{f}}^{\left(1\right)}+{\mathbf{f}}^{\left(2\right)}$$

where the strain–displacement relation $\mathbf{e}=\frac{1}{2}\left(\nabla \mathbf{u}+\mathbf{u}\nabla \right)$ is considered in Eq. (2). The following nondimensional parameters can be introduced:

3 $$\begin{aligned} & {{\varvec{\upchi}}} = {\mathbf{I}} - \frac{{{\mathbf{u}}^{\left( 2 \right)} }}{{{\mathbf{u}}^{\left( 1 \right)} }} \\ & {{\varvec{\upxi}}} = {\mathbf{I}} - \frac{{\lambda^{\left( 2 \right)} {\mathbf{u}}^{\left( 2 \right)} }}{{\lambda^{\left( 1 \right)} {\mathbf{u}}^{\left( 1 \right)} }} = {\mathbf{I}} - \frac{{\lambda^{\left( 2 \right)} \left( {{\mathbf{I}} - {{\varvec{\upchi}}}} \right)}}{{\lambda^{\left( 1 \right)} }} \\ & {\varvec{\epsilon}} = {\mathbf{I}} - \frac{{{\mathbf{C}}^{\left( 2 \right)} }}{{{\mathbf{C}}^{\left( 1 \right)} }} \\ \end{aligned}$$

where ${\varvec{\upchi}}$ is a measure of the material’s asymmetric deformation, ${\varvec{\upxi}}$ is a measure of the asymmetry in the material’s dynamic response, ${\varvec{\epsilon}}$ is a measure of the asymmetry in the material’s elasticity, which indicates a material with nonreciprocal elasticity. Employing the nondimensional parameters in Eq. (3), Eq. (2) can be rewritten in the form:

4 $$\nabla \cdot \left(\frac{1}{2}{\mathbf{C}}^{\left(1\right)}:\left(\nabla {\mathbf{u}}^{\left(1\right)}+{\mathbf{u}}^{\left(1\right)}\nabla \right)\right)+\rho {\lambda }^{\left(1\right)}{\mathbf{u}}^{\left(1\right)}-\nabla \cdot \left(\frac{1}{2}{\mathbf{C}}^{\left(1\right)}:\left(\nabla {\mathbf{u}}^{\left(1\right)}+{\mathbf{u}}^{\left(1\right)}\nabla \right)\right)\left(\mathbf{I}-{\varvec{\upepsilon}}\right)\left(\mathbf{I}-{\varvec{\upchi}}\right)-\rho {\lambda }^{\left(1\right)}{\mathbf{u}}^{\left(1\right)}\left(\mathbf{I}-{\varvec{\upxi}}\right)=-{\mathbf{f}}^{\left(1\right)}+{\mathbf{f}}^{\left(2\right)}$$

As previously demonstrated, a material with an effective nonreciprocity is the one that gives asymmetric responses when tested under the same kinematical and kinetical conditions. Therefore, when the two forces ${\mathbf{f}}^{\left(1\right)}$ and ${\mathbf{f}}^{\left(2\right)}$ have the same magnitude but two opposite directions (i.e., $-{\mathbf{f}}^{\left(1\right)}+{\mathbf{f}}^{\left(2\right)}={\mathbf{0}}$), the material’s static nonreciprocity (${{\varvec{\upchi}}}_{s}$) and dynamic nonreciprocity (${{\varvec{\upxi}}}_{d}$) can be obtained from Eq. (4), as follows:

5 $${{\varvec{\upchi}}}_{s}=\frac{{\varvec{\epsilon}}}{{\varvec{\epsilon}}-\mathbf{I}}$$

6 $${{\varvec{\upxi}}}_{d}=-\frac{\nabla \cdot \left(\frac{1}{2}{\mathbf{C}}^{\left(1\right)}:\left(\nabla {\mathbf{u}}^{\left(1\right)}+{\mathbf{u}}^{\left(1\right)}\nabla \right)\right)\left({\varvec{\epsilon}}\left(\mathbf{I}-{\varvec{\upchi}}\right)+{\varvec{\upchi}}\right)}{\rho {\lambda }^{\left(1\right)}{\mathbf{u}}^{\left(1\right)}}$$

It should be noted that the search for the static nonreciprocity requires, by definition, $\rho {\lambda }^{\left(1\right)}{\mathbf{u}}^{\left(1\right)}={\mathbf{0}}$.

It follows from Eq. (5) that the static nonreciprocity (${{\varvec{\upchi}}}_{s}$) is achieved only and only if the material has nonreciprocal elasticity (i.e., ${\varvec{\epsilon}}\ne {\mathbf{0}}$). This indicates that the static nonreciprocity is not associated with the material nonlinearity, but, definitely, it is associated with the material’s nonreciprocal elasticity.

Equation (6) indicates that the dynamic nonreciprocity of passive materials can be achieved if the material can exhibit either asymmetric deformation (${\varvec{\upchi}}$) or nonreciprocal elasticity (${\varvec{\epsilon}}$). A nonlinear material can exhibit asymmetric deformation (${\varvec{\upchi}}$) only under two different kinetical and kinematical conditions, and—in this case—a reciprocal elastic (${\varvec{\epsilon}}={\mathbf{0}}$) material can achieve dynamic nonreciprocity. However, under the same kinematical and kinetical conditions, the dynamic nonreciprocity is conditional by the existence of a nonreciprocal elasticity.

### Metamaterial with nonreciprocal elasticity

**Figure 3:**
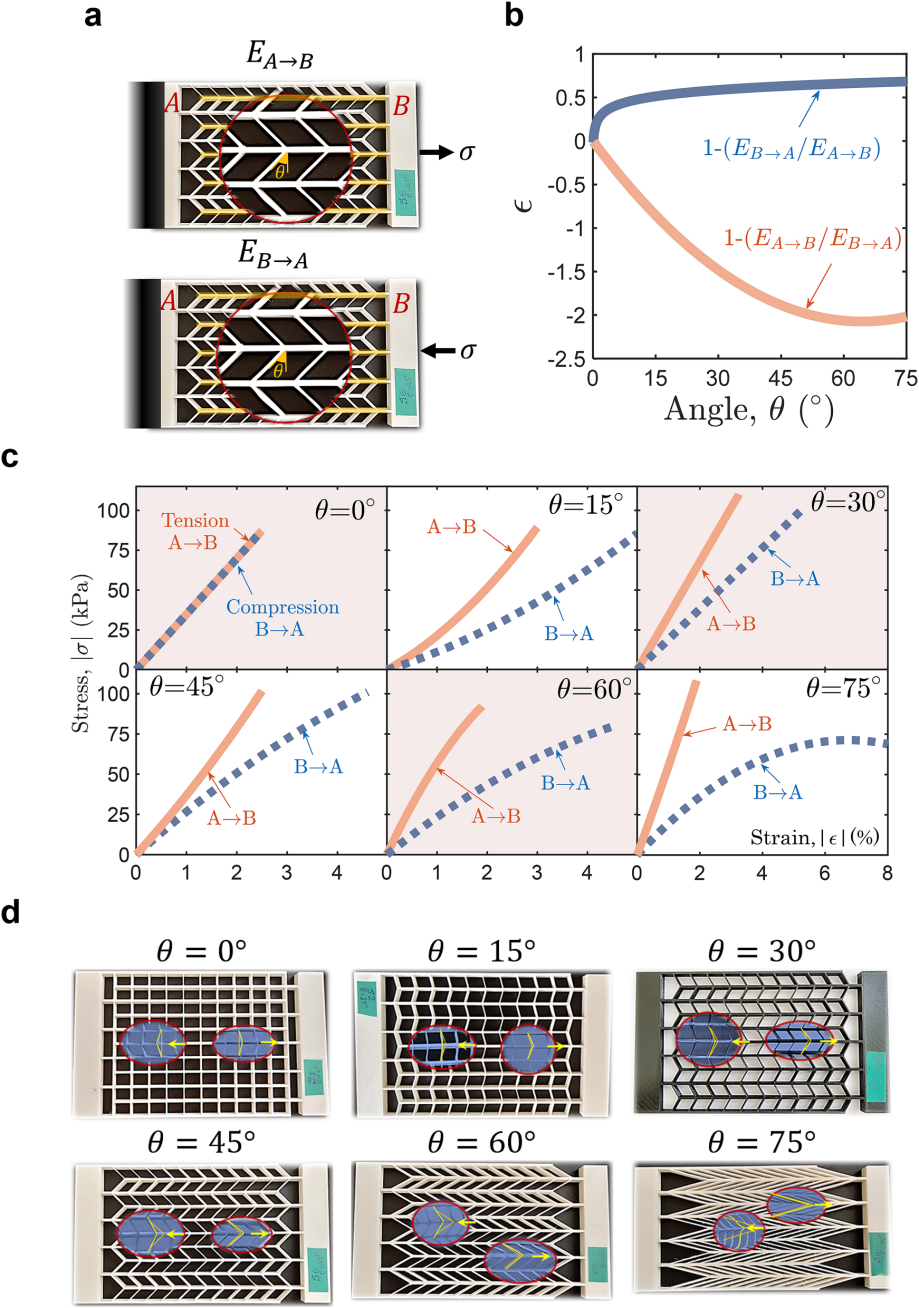
Metamaterials with nonreciprocal linear and nonlinear elasticities. (**a**) One sample of the developed metamaterial with a geometrical angle $\theta =45^\circ$. The material sample is tested under tension and compression such that it is fixed at side A and loaded from side B. Under the tensile test, the tensile load is applied on the highlighted ribs, which are pulled to the right, $A\to B$. Under the compression test, the compressive load is applied on the same ribs, which are pushed to the left, $B\to A$. (**b**) The nonreciprocal elasticity parameter $\epsilon$ versus the geometrical angle $\theta$ (experimental results). (**c**) The elastic stress–strain curves of the tensile tests ($A\to B$) and compression tests ($B\to A$) of the 3D printed material samples with different geometrical angles $\theta =0^\circ ,15^\circ ,30^\circ ,45^\circ ,60^\circ ,75^\circ$. (**d**) Images of the 3D printed material samples. The insets show the asymmetric deformations of the material under tension and compression.

Here, we demonstrate the *nonreciprocal elasticity* experimentally. We developed a metamaterial with a special periodic microstructure to achieve nonreciprocal elasticity (Fig. 3a). The periodic microstructure of the developed metamaterial consists of cross-linked elastic ribs. The struts that link the ribs are tilted with respect to the ribs’ normal with an angle, $\theta$ (see Fig. 3a). The angle, $\theta$, is a geometrical parameter of the metamaterial that gave the control of the degree of the nonreciprocal elasticity achieved by the metamaterial.

Six samples of the metamaterial with different geometrical angles, $\theta =0^\circ ,15^\circ ,30^\circ ,45^\circ ,60^\circ$, and $75^\circ$ were 3D-printed and tested under tension and compression (Fig. 3a). All material samples were 3D printed by Fused-Deposition Modeling (FDM) using *Felix Pro 3-Touch* 3D printer. All samples (except the material sample with $\theta =30^\circ$) were 3D printed from Thermoplastic Polyester Elastomer (TPE) filament (*FilaFlexible40*) with 10 MPa tensile modulus and > 400% elongation. Each material sample was tested under tension ($A\to B$) and compression ($B\to A$) according to the setup shown in Fig. 3a.The material samples were tested up to a specific elongation %, and the stress–strain curves were obtained. The TPE filament gave material samples with nonlinear elastic stress–strain curves. To show that nonreciprocal elasticity can be realized by linear elastic materials, the material sample with $\theta =30^\circ$ was 3D printed from Thermoplastic Polyurethane (TPU) filament with 150 MPa tensile modulus and 450% elongation. The TPU filament gave a material with linear elastic stress–strain curves. The experimental results of the tensile and compression tests of the various material samples are represented in Fig. 3b,c. The 3D printed material samples are shown in Fig. 3d.

Tailoring the geometrical angle, $\theta$, gave us the ability to tailor the degree of the material’s nonreciprocal elasticity. We defined, $\epsilon$, as a nonreciprocal elasticity measure:

7 $$\epsilon =1-\frac{{E}_{B\to A}}{{E}_{A\to B}}\;{\mathrm{or}}\;1-\frac{{E}_{A\to B}}{{E}_{B\to A}}$$

where ${E}_{A\to B}$ (${E}_{B\to A}$) is the material’s elastic modulus measured by the tension (compression) test such that the applied load was directed from A (B) to B (A). When $\epsilon =0$, the material is reciprocal elastic and ${E}_{B\to A}={E}_{A\to B}$. When $\epsilon \ne 0$, the material exhibits nonreciprocal elasticity. A typical range of the nonreciprocal elasticity parameter is $-\infty <\epsilon <1$. Figure 3b shows the experimental results of the variation of the nonreciprocal elasticity parameter, $\epsilon$, as a function of the geometrical angle, $\theta$.

When $\theta =0^\circ$, the metamaterial is reciprocal elastic ($\epsilon =0$) where only one stress–strain curve was obtained when it is tested under the two setups; tension ($A\to B$) and compression ($B\to A$) (Fig. 3c). This material exhibited the same elasticity no matter what the direction of the material sample is with respect to the applied stress direction. However, the other material samples with geometrical angles $\theta >0$ showed a robust stress direction dependence, and exhibited asymmetric deformations and significant nonreciprocal elasticities ($\epsilon \ne 0$). Two different stress–strain curves were obtained when testing the material under the two experimental setups ($A\to B$ and $B\to A$). In the tensile test (setup $A\to B$), the material showed a strong stiffness against the applied tensile stress. In contrast, it appeared of a lower stiffness in the compression test (setup $B\to A$). The contrast in the material stiffnesses can be attributed to the geometrical asymmetry that has been achieved by tilting the struts with respect to the horizontal ribs. The nonreciprocal elasticity increased due to an increase in the geometrical angle $\theta$ (Fig. 3b).

It follows from Fig. 3c that the asymmetry in the material deformation and the nonreciprocity can be achieved using linear elastic materials. We tested a material sample, which was intentionally 3D printed of a filament with a higher elastic modulus (TPU filament), to give two different linear elastic stress–strain curves ($\theta =30^\circ$) at the same applied load range used for the other samples. Despite this material sample is linear elastic, it exhibited a significant nonreciprocal elasticity. These results indicate that the attainment of the nonreciprocity that depends on breaking the symmetry in the material deformation is possible using linear materials if a suitable microstructural-geometrical asymmetry is crafted into the material. In addition, the nonlinearity is not the only condition for achieving asymmetric deformation of the material or to realize mechanical nonreciprocity. Nonetheless, concurrently achieving nonlinearity and microstructural asymmetries promotes the mechanical nonreciprocity^7,8,10,45–47^.

### Nonreciprocal elasticity and topological mechanics

**Figure 4:**
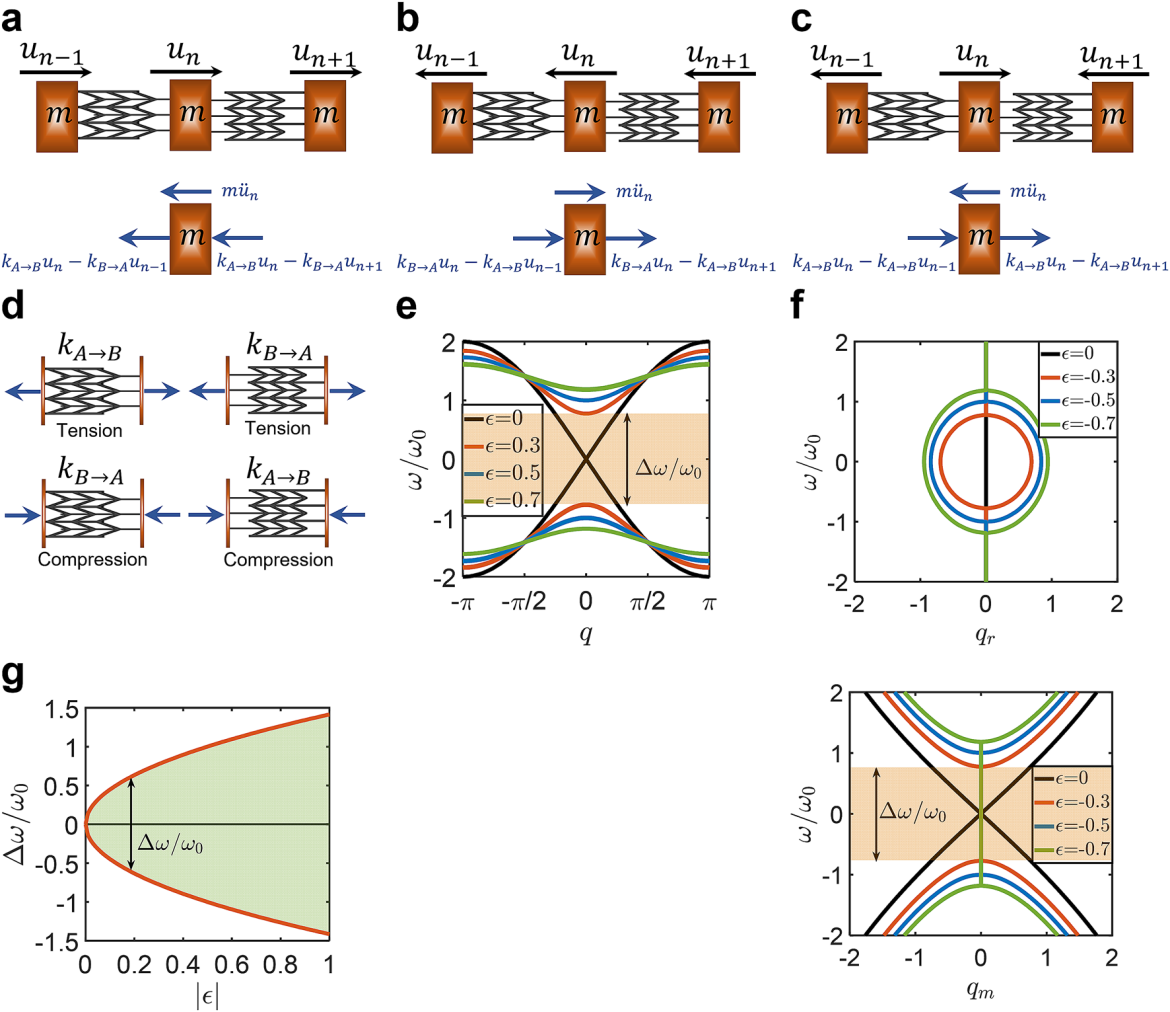
Topological mechanics of monoatomic lattices with nonreciprocal elastic springs. (**a**–**c**) Three configurations of a mass-spring system representing a monoatomic lattice ($m$ indicates the atomic mass). Nonreciprocal springs made of the developed metamaterial are considered such that the stiffness is different when the spring is stretched/compressed from two opposite ends (i.e., ${k}_{A\to B}>{k}_{B\to A}$). The free body diagrams of the spring forces and the inertia forces are represented when (**a**) atoms vibrate to the right, (**b**) when atoms vibrate to the left, and (**c**) when each two neighbor atoms vibrate in two opposite directions. (**d**) Representations of the different stiffnesses of the considered nonreciprocal springs under tension and compression. (**e**) Classical band structures $\omega \left(q\right)$ (nondimensional frequency ($\omega /{\omega }_{0}$) versus nondimensional wavenumber ($q$)) are obtained when atoms vibrate to the right with positive nonreciprocal elasticity ($\epsilon >0$). (**f**) Complex band structures are obtained when atoms vibrate to the left with negative nonreciprocal elasticity ($\epsilon <0$) (real band structures $\omega \left({q}_{r}\right)$ (above) and imaginary band structures $\omega \left({q}_{m}\right)$ (below)). (**g**) The band-gap ($\Delta \omega$) versus the nonreciprocal elasticity parameter ($\epsilon$).

**Figure 5:**
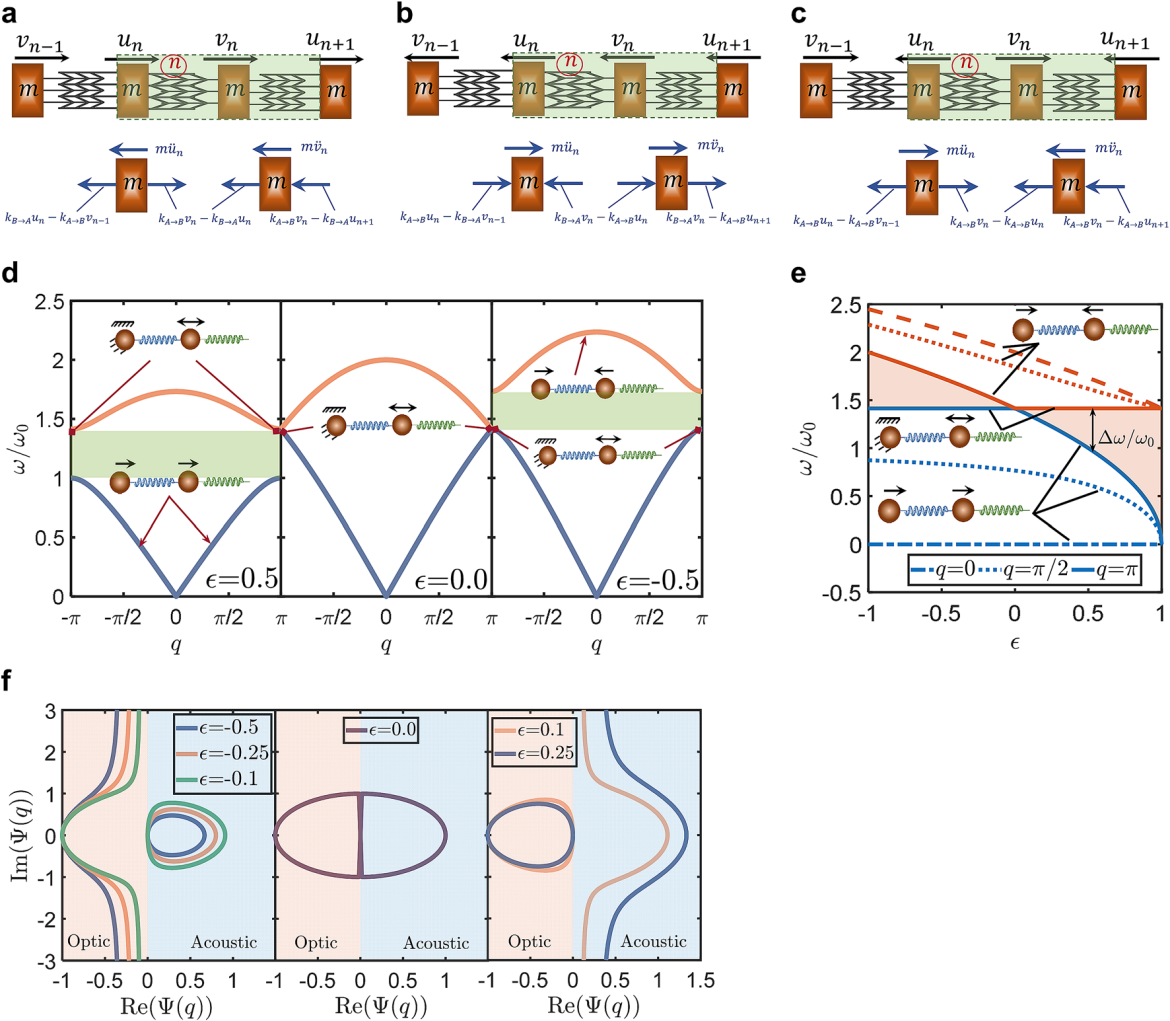
Topological Mechanics of Diatomic Lattices with Nonreciprocal Elastic Springs. (**a**–**c**) Three configurations of a diatomic lattice model. Nonreciprocal springs made of the developed metamaterial are considered such that the stiffness is different when the spring is stretched/compressed from two opposite ends (${k}_{A\to B}>{k}_{B\to A}$). The free body diagrams of the spring forces and the inertia forces are represented when (**a**) atoms vibrate to the right, (**b**) when atoms vibrate to the left, and (**c**) when each atom vibrates opposite to its two neighbor-atoms. (**d**) The band structure $\omega \left(q\right)$ of the diatomic lattice for different values of the nonreciprocal elasticity parameter, $\epsilon =0.5$ (left), $\epsilon =0$ (middle), and $\epsilon =-0.5$ (right). (**e**) The acoustic and optical frequencies at different wavenumbers, $q=0,\pi /2$ and $\pi$, versus the nonreciprocal elasticity parameter $\epsilon$. The insets represent the Floquet–Bloch eigenmodes. (**f**) Contour plots of the acoustic and optic eigenmodes $\Psi \left(q\right)$ in the complex plan for different values of the nonreciprocal elasticity parameter $\epsilon$.

By means of topological mechanics, we further demonstrate that the trigger of the static mechanical nonreciprocity is a “nonreciprocal elasticity”. We investigated the topological mechanics of monoatomic and diatomic lattices. The conventional reciprocal springs of these lattices were replaced by nonreciprocal springs made of the proposed metamaterial (Figs. 4 and 5). Implementing the proposed metamaterial gave the spring stiffness to a stretch/compression from one end is different than the spring stiffness to a stretch/compression from the other end, i.e., ${k}_{A\to B}\ne {k}_{B\to A}$ (Figs. 4 and 5). The measure of the nonreciprocal elasticity of these springs ($\epsilon$) was defined, as given in Eq. (7).

#### Topological mechanics of monoatomic lattices with nonreciprocal elastic springs

Figure 4 shows a mass-spring system of a monoatomic lattice with linear springs. The springs are of nonreciprocal elasticity such that the spring stiffness to a stretch/compression from one end is different than the stiffness to a stretch/compression from the other end, i.e., ${k}_{A\to B}>{k}_{B\to A}$ (see Fig. 4d for the different stiffnesses of the nonreciprocal springs under tension and compression). The three possible configurations of the vibration of the monoatomic lattice are considered (Fig. 4a–c). In the configuration shown in Fig. 4a,b, all atoms vibrate to the right (left). In addition, one configuration is considered such that atom $n$ vibrates in a direction that is opposite to the direction of vibration of the two neighbor atoms (Fig. 4c).

First, the static nonreciprocity of monoatomic lattices with nonreciprocal springs (Fig. 4) is demonstrated by determining the elastostatic deformations when the lattice is subjected to a static force $F$. Consider the lattice such that atom $n+1$ is fixed (i.e., ${u}_{n+1}=0$) and the force is applied on atom $n-1$. Referring to the free body diagrams represented in Fig. 4a,b, the equilibrium equation of the atom $n$ was determined, and the difference in the atom displacement upon switching the direction of the applied force was obtained by:

8 $$\Delta {u}_{n}=\left|\frac{F}{{k}_{A\to B}}-\frac{F}{{k}_{B\to A}}\right|$$

It is clear that the monoatomic lattice with nonreciprocal springs exhibits different displacements upon switching the direction of the applied force (i.e., $\Delta {u}_{n}>0$). If reciprocal springs are used (i.e., ${k}_{A\to B}={k}_{B\to A}$), the monoatomic lattice exhibits a symmetric deformation (i.e., $\Delta {u}_{n}=0$).

Second, we studied the emerging topological properties of monoatomic lattices with nonreciprocal springs at the three different configurations (Fig. 4a–c). According to Newton’s second law of the dynamic equilibrium, the equations of motion of atom $n$ can be expressed for the different configurations, as follows:

9 $$\begin{aligned} & Configuration \, a \\ & m\ddot{u}_{n} + 2k_{A \to B} u_{n} - k_{B \to A} \left( {u_{n - 1} + u_{n + 1} } \right) = 0 \\ & Configuration \, b \\ & m\ddot{u}_{n} + 2k_{B \to A} u_{n} - k_{A \to B} \left( {u_{n - 1} + u_{n + 1} } \right) = 0 \\ & Configuration \, c \\ & m\ddot{u}_{n} + 2k_{A \to B} u_{n} - k_{A \to B} \left( {u_{n - 1} + u_{n + 1} } \right) = 0 \\ \end{aligned}$$

where ${u}_{n}$, ${u}_{n-1}$, and ${u}_{n+1}$ are the displacements of atoms $n$, $n-1$, and $n+1$, respectively. $m$ is the mass of the atom. ${k}_{A\to B}$ and ${k}_{B\to A}$ are the two stiffnesses of the nonreciprocal spring. The dot denotes a time derivative.

Assume an infinite lattice, the atomic displacement can be expressed—using a Floquet–Bloch expansion—as follows:

10 $${u}_{n}=U\mathrm{exp}\left(i\left(nq-\omega t\right)\right)$$

where $\omega$ is the frequency, and $q$ is the nondimensional wavenumber. The substitution of Eq. (10) into Eq. (9) gives the following dispersion relations for the different configurations (${k}_{A\to B}>{k}_{B\to A}$):

11 $${\omega }^{2}-{\omega }_{0}^{2}\left(2-2\left(1-\epsilon \right){\mathrm{cos}}\;q\right)=0$$

where $\epsilon$ is the nonreciprocal elasticity parameter, and ${\omega }_{0}$ is a fundamental frequency of the lattice. For *Configuration a* (*b*), ${\omega }_{0}=\sqrt{{k}_{A\to B}/m}$ (${\omega }_{0}=\sqrt{{k}_{B\to A}/m}$) and $\epsilon =1-\left({k}_{B\to A}/{k}_{A\to B}\right)$ ($\epsilon =1-\left({k}_{A\to B}/{k}_{B\to A}\right)$). For *Configuration c*, ${\omega }_{0}=\sqrt{{k}_{A\to B}/m}$ and $\epsilon =0$.

The band-gap width $\Delta \omega =\omega \left(q=0\right)$ of the different configurations can be obtained according to Eq. (11) in the form:

12 $$\Delta \omega =2{\omega }_{0}\sqrt{2\left|\epsilon \right|}$$

The expression of the band-gap width is the same for *Configurations a* and *b*. However, the band-gap width vanishes for *Configuration c* because $\epsilon =0$.

The monoatomic lattice shown in Fig. 4 exhibits different elasticities and band structures when its atoms are displaced in different directions while vibrating. When all atoms vibrate to the right (left), the nonreciprocal elasticity parameter is positive (negative) such that $\epsilon =1-\left({k}_{B\to A}/{k}_{A\to B}\right)$ ($\epsilon =1-\left({k}_{A\to B}/{k}_{B\to A}\right)$). According to Eq. (11), the responsive behavior of the monoatomic lattice with positive nonreciprocal elasticity ($\epsilon >0$) is described by a classical band structure (Fig. 4e). However, when the lattice exhibits a negative nonreciprocal elasticity ($\epsilon <0$), a complex band structure within the real frequency domain is obtained (Fig. 4f). Complex band structures can be obtained by solving Eq. (11) for the wavenumber, $q$, for a given frequency, $\omega$, which shall be complex for all given real frequencies^48,49^. Therefore, $q={q}_{r}+i{q}_{m}$ is substituted into Eq. (11), which gives (note the changes in the signs of the terms to arbitrary postulate real frequencies):

13 $$\mathrm{cos}\left({q}_{r}+i{q}_{m}\right)=\frac{{\omega }^{2}}{2{\omega }_{0}^{2}\left(1-\epsilon \right)}+\frac{1}{1-\epsilon }$$

where ${q}_{r}$ and ${q}_{m}$ are the real and complex parts of the wavenumber. Considering that ${\mathrm{cos}}\;\left({q}_{r}+i{q}_{m}\right)={\mathrm{cos}}\;{q}_{r}\;{\mathrm{cosh}}\;{q}_{m}+i\;{\mathrm{sin}}\;{q}_{r}\;{\mathrm{sinh}}{q}_{m}$, the following two relations are obtained:

14 $${\mathrm{cos}}\;{q}_{r}\;{\mathrm{cosh}}\;{q}_{m}=\frac{{\omega }^{2}}{2{\omega }_{0}^{2}\left(1-\epsilon \right)}+\frac{1}{1-\epsilon }$$

15 $${\mathrm{sin}}\;{q}_{r}\;{\mathrm{sinh}}\;{q}_{m}=0$$

According to Eq. (15), either ${q}_{r}=n\pi$ or ${q}_{m}=0$, where $n=\mathrm{0,1},2, \dots$.

The real part of the wavenumber is obtained by setting ${q}_{m}=0$ (thus, ${\mathrm{cos}}\;q={\mathrm{cos}}\;{q}_{r}$) in Eq. (14), which gives:

16 $${q}_{r}={\mathrm{cos}}^{-1}\left(\frac{{\omega }^{2}}{2{\omega }_{0}^{2}\left(1-\epsilon \right)}+\frac{1}{1-\epsilon }\right)$$

On the other hand, the complex part of the wavenumber is obtained by setting ${q}_{r}=n\pi$ (thus, ${\mathrm{cos}}\;q={\mathrm{cosh}}\;{q}_{m}$ for ${q}_{r}=\mathrm{0,2}\pi ,4\pi ,\dots$, and ${\mathrm{cos}}\;q=-{\mathrm{cosh}}\;{q}_{m}$ for ${q}_{r}=\pi ,3\pi ,\dots$) in Eq. (14), which gives:

17 $$q_{m} = \left\{ {\begin{array}{*{20}l} {{\text{cosh}}^{{ - 1}} \left( {\frac{{\omega ^{2} }}{{2\omega _{0}^{2} \left( {1 - \epsilon} \right)}} + \frac{1}{{1 - \epsilon}}} \right)} \hfill & {if\;q_{r} = {\text{0}},{\text{2}}\pi ,4\pi , \ldots } \hfill \\ {{\text{cosh}}^{{ - 1}} \left( { - \frac{{\omega ^{2} }}{{2\omega _{0}^{2} \left( {1 - \epsilon} \right)}} - \frac{1}{{1 - \epsilon}}} \right)} \hfill & {if\;q_{r} = \pi ,3\pi ,5\pi , \ldots } \hfill \\ \end{array} } \right.$$

The classical and the complex band structures of the two *Configurations a* and *b* of the monoatomic lattice are represented in Fig. 4e,f, respectively. Gaps are observed in the obtained band structures where the band-gap width ($\Delta \omega$) robustly depends on the elastic nonreciprocity of the springs (Fig. 4g). The same gap structure was obtained for the classical and complex bands. Nonetheless, inside the gap of the complex band, the real part of the wavenumber is non-zero while the imaginary part is zero. A non-zero real wavenumber within the band-gap indicates an evanescent wave^49^. Thus, the responsive behavior of the monoatomic lattice of $\epsilon <0$ within the band-gap is an exponentially localized wave centered at the excitation point and evanescently decays on both sides^6,49^. The band-gap vanishes if and only if the lattice is reciprocal elastic such that $\epsilon =0$. This occurs when the atoms of the lattices take the *Configuration c* during the vibration.

The existence of a vanishing gap when the lattice is reciprocal elastic (i.e., $\epsilon =0$) indicates that the emerging topology of monoatomic lattices with nonreciprocal springs is non-trivial^7,50,51^. In addition, the two *Configurations a* and *b* of the lattice are topologically distinct (one configuration revealed a classical band structure while the other revealed a complex band structure). It is also interesting to observe that the transformation between these two distinct topologies requires closing the gap (in other words, it requires reciprocal elasticity). This indicates that the transformation of one configuration to the other requires an intermediate configuration at which the lattice reveals reciprocal elasticity ($\epsilon =0$). This intermediate configuration is when each atom vibrates opposite to its two neighbor-atoms (the middle atom vibrates in a direction that is opposite to the vibration of the other two atoms) (*Configuration c*). These topological properties of the monoatomic lattice with nonreciprocal springs demonstrate that the trigger of the static mechanical nonreciprocity is a nonreciprocal elasticity.

#### Topological mechanics of diatomic lattices with nonreciprocal elastic springs

Now, let us consider the diatomic lattice model shown in Fig. 5. The unit cell contains two atoms of equal masses, $m$, and two springs with nonreciprocal elasticity. The springs are arranged as given in Fig. 5. The possible configurations of the vibration of the diatomic lattice are three, which are considered as shown in Fig. 5a–c.

The nonreciprocity of the diatomic lattice considered in Fig. 5 is demonstrated by deriving the elastostatic deformation of the lattice when subjected to a static force $F$. The lattice is considered such that one end is fixed while the other end is subjected to the force $F$. Referring to the free body diagrams represented in Fig. 5a,b, the equilibrium equations of the atoms that belong to the unit cell $n$ were determined, and the difference in the atomic displacements upon switching the direction of the applied force were obtained as follows:

18 $$\begin{gathered} \Delta u_{n} = \left| {\frac{2F}{{k_{A \to B} }} - \frac{2F}{{k_{B \to A} }}} \right| \hfill \\ \Delta v_{n} = \left| {\frac{F}{{k_{A \to B} }} - \frac{F}{{k_{B \to A} }}} \right| \hfill \\ \end{gathered}$$

It is clear that the diatomic lattice with nonreciprocal springs exhibits different displacements upon switching the direction of the applied force (i.e., $\Delta {u}_{n}>0$ and $\Delta {v}_{n}>0$). If reciprocal springs are used (i.e., ${k}_{A\to B}={k}_{B\to A}$), the diatomic lattice exhibits a symmetric deformation (i.e., $\Delta {u}_{n}=\Delta {v}_{n}=0$).

We also studied the topological properties of diatomic lattices with nonreciprocal elastic springs. The topological properties of the three different configurations of the diatomic lattice were determined. According to Newton’s second law of the dynamic equilibrium, the equations of motion of the two atoms that belong to the unit cell $n$ can be expressed for *Configuration a* (Fig. 5a), as follows:

19 $$m{\ddot{u}}_{n}+2{k}_{B\to A}{u}_{n}-{k}_{A\to B}\left({v}_{n}+{v}_{n-1}\right)=0$$

20 $${\ddot{v}}_{n}+2{k}_{A\to B}{v}_{n}-{k}_{B\to A}\left({u}_{n}+{u}_{n+1}\right)=0$$

For *Configuration b* (Fig. 5b), the equations of motion become:

21 $$m{\ddot{u}}_{n}+2{k}_{A\to B}{u}_{n}-{k}_{B\to A}\left({v}_{n}+{v}_{n-1}\right)=0$$

22 $$m{\ddot{v}}_{n}+2{k}_{B\to A}{v}_{n}-{k}_{A\to B}\left({u}_{n}+{u}_{n+1}\right)=0$$

and for *Configuration c* (Fig. 5c):

23 $$m{\ddot{u}}_{n}+2{k}_{A\to B}{u}_{n}-{k}_{A\to B}\left({v}_{n}+{v}_{n-1}\right)=0$$

24 $$m{\ddot{v}}_{n}+2{k}_{A\to B}{v}_{n}-{k}_{A\to B}\left({u}_{n}+{u}_{n+1}\right)=0$$

where $u$ and $v$ are the independent displacements of the two atoms. The subscripts $n$, $n-1$, and $n+1$ refer to the unit cells $n$, $n-1$, and $n+1$, respectively. ${k}_{A\to B}$ and ${k}_{B\to A}$ are the two stiffnesses of the nonreciprocal springs (refer to Fig. 4d for more details).

Assume an infinite lattice, the atomic displacements, $u$ and $v$, can be expressed—using a Floquet–Bloch expansion—as follows:

25 $${u}_{n}=U\mathrm{exp}\left(i\left(nq-\omega t\right)\right)$$

26 $${v}_{n}=V\mathrm{exp}\left(i\left(nq-\omega t\right)\right)$$

where $\omega$ is the frequency, and $q$ is the nondimensional wavenumber. The substitution of Eqs. (25) and (26) into Eqs. (19) and (20) gives the equations of motion of *Configuration a* in the form:

27 $$\left[\begin{array}{cc}2{\omega }_{B}^{2}-{\omega }^{2}& -{\omega }_{A}^{2}\left(1+\mathrm{exp}\left(-iq\right)\right)\\ -{\omega }_{B}^{2}\left(1+\mathrm{exp}\left(iq\right)\right)& 2{\omega }_{A}^{2}-{\omega }^{2}\end{array}\right]\left\{\begin{array}{c}U\\ V\end{array}\right\}=\left\{\begin{array}{c}0\\ 0\end{array}\right\}$$

where ${\omega }_{A}=\sqrt{{k}_{A\to B}/m}$ and ${\omega }_{B}=\sqrt{{k}_{B\to A}/m}$. The determinant of the coefficient matrix gives the dispersion relation, as follows:

28 $${\omega }^{2}={\omega }_{A}^{2}\left(2-\epsilon \pm \sqrt{1+{\left(1-\epsilon \right)}^{2}+2\left(1-\epsilon \right){\mathrm{cos}}\;q}\right)$$

where $\epsilon =1-\left({k}_{B\to A}/{k}_{A\to B}\right)$ is the nonreciprocal elasticity parameter. Because ${k}_{A\to B}>{k}_{B\to A}$, the nonreciprocal elasticity parameter $\epsilon$ is positive for *Configuration a*.

Similarly, the dispersion relation for *Configuration b* can be obtained, as follows:

29 $${\omega }^{2}={\omega }_{B}^{2}\left(2-\epsilon \pm \sqrt{1+{\left(1-\epsilon \right)}^{2}+2\left(1-\epsilon \right){\mathrm{cos}}\;q}\right)$$

where the nonreciprocal elasticity parameter is negative and has the form $\epsilon =1-\left({k}_{A\to B}/{k}_{B\to A}\right)$.

The dispersion relation for *Configuration c* can be obtained by setting $\epsilon =0$ in Eqs. (28) or (29) where the model reduces to the classical diatomic lattice with identical masses and springs^49^.

The band-gap width $\Delta \omega =\omega \left(q=\pi \right)$ can be obtained according to Eqs. (28) and (29), as follows:

30 $$\Delta \omega = \left\{ {\begin{array}{*{20}l} {\omega _{A} \left( {\sqrt 2 - \sqrt {2 - 2\epsilon} } \right)} \hfill & {if\quad\epsilon > 0\quad Configuration\;a} \hfill \\ {\omega _{B} \left( {\sqrt {2 - 2\epsilon} - \sqrt 2 } \right)} \hfill & {if\quad\epsilon < 0\quad Configuration\;b} \hfill \\ \end{array} } \right.$$

To reveal the band structures of the diatomic lattice at the three configurations, we present in Fig. 5d the band structures of the diatomic lattice for cases when $\epsilon =0.5$, $0$, and $-0.5$. Band-gaps ($\Delta \omega$) are noticeably seen in the band structures as long as $\epsilon \ne 0$ (i.e., $\Delta \omega ={\omega }_{0}\left|\sqrt{2}-\sqrt{2-2\epsilon }\right|$). Whereas the band structures of the diatomic lattice obtained for positive and negative nonreciprocal elasticities ($\epsilon =0.5$ and $-0.5$) would look similar, they are—in fact—different in the band-gap structure and the evolution of the eigenmodes. We observed “Eigenvalue Loci Veering” due to a change in the nonreciprocal elasticity (Fig. 5e). The acoustic and optical frequencies approach each other and then veer apart as $\epsilon$ changes from positive to negative. The transition from “approaching” to “veering” of the acoustic and optical frequencies takes place exactly at $\epsilon =0$ (where the gap is completely closed). This interesting observation of the eigenvalue loci veering—which takes place only and only if the lattice is reciprocal elastic—indicates a non-trivial topology. In other words, the eigenvalue loci veering signifies the transition between two distinct topologies.

The eigenvalue loci veering is mainly due to a rapid variation in the eigenvectors, which would result in either a mode inversion^52^ (band inversion^53^) or a mode localization^54,55^ (band localization). This is further investigated in Fig. 5f, by observing the evolution of the acoustic and optic eigenvectors $${\left\{\begin{array}{cc}1&\Psi \left(q\right)\end{array}\right\}}^{\mathrm{T}}$$. We studied the variation of the acoustic and optic eigenvectors with the nonreciprocal elasticity parameter $\epsilon$. According to Eqs. (27)–(29), we obtained the acoustic and optic eigenvectors for *Configurations a* and *b* with the form:

31 $${\varvec{\Psi}}=\left\{\begin{array}{c}1\\ \Psi \left(q\right)\end{array}\right\}$$

where

32 $$\Psi \left(q\right)=\frac{C\left(q\right)\sqrt{2\left(1+{\mathrm{cos}}\;q\right)}}{\left|C\left(q\right)\right|\left(-\epsilon \mp \sqrt{{\epsilon }^{2}+2\left(1-\epsilon \right)\left(1-{\mathrm{cos}}\;q\right)}\right)}$$

where $C\left(q\right)=1+\mathrm{exp}\left(-iq\right)$ is a term that depends on the off-diagonal component of the dynamical matrix in Eq. (27). $\left|C\left(q\right)\right|$ is the magnitude of this complex term ($C\left(q\right)$). For *Configuration a* (*b*), $\epsilon$ is positive (negative). The acoustic and optic eigenvectors for *Configuration c* are obtained by setting $\epsilon =0$ in Eq. (32).

It follows from Fig. 5e,f that the acoustic and optic frequencies and mode shapes are distinct when $0<q<\pi$. The acoustic mode is in-phase where $\mathrm{Re}\left(\Psi \left(q\right)\right)>0$, while the optical mode is out-of-phase where $\mathrm{Re}\left(\Psi \left(q\right)\right)<0$. The eigenvalue loci veering takes place when $q=\pi$, and it causes mode (or band) localization without band inversion. The contours of the acoustic (optic) eigenmodes are confined to the right (left) of the complex plane (see Fig. 5f and the insets in Fig. 5e). This indicates no winding about the origin and no band inversion. A closed contour indicates a band localization due to the eigenvalue loci veering where the vibration energy is inhibited to be localized at only one atom of the diatomic lattice (Fig. 5f). When $\epsilon =0$, the acoustic and optic contours are closed, which indicates that the acoustic and optic bands have the same frequency and mode shape, and are localized when $q=\pi$, i.e., $\mathrm{Re}\left(\Psi \left(q\right)\right)=0$ and $\mathrm{Im}\left(\Psi \left(q\right)\right)=0$. When $\epsilon \ne 0$, the band localization occurs for either acoustic or optic bands depending on the sign of $\epsilon$. In *Configuration a* of the diatomic lattice (Fig. 5a) where $\epsilon >0$, the optic contour is closed and indicates band localization. On the other hand, the acoustic contour is open and indicates no band localization where the vibration energy distributes between the two atoms. In *Configuration b* (Fig. 5b) where $\epsilon <0$, acoustic and optical bands switch roles, where acoustic bands are localized (see Fig. 5f).

These observed differences in the eigenmodes of the acoustic and optic bands are associated with a band-gap formation. This demonstrates that the formation of band-gaps in the band structure of diatomic lattices with nonreciprocal springs requires a localization of either the acoustic energy or the optical energy at only one atom. However, no band-gaps are formed if both acoustic and optical energies are localized. Generally speaking, the formation of the band-gap of a topological mechanical system would require a confinement of the vibration energy to one of the bands, which can be achieved if a proper nonreciprocal elasticity is implemented.

It also follows from Fig. 5f that the two *Configurations a* and *b* of the diatomic lattice are topologically distinct, and the transformation between these two distinct topologies requires a reciprocal elasticity (requires closing the gap). Similar to the monoatomic lattice, when the diatomic lattice transforms between the two configurations, it exhibits an intermediate configuration at which each atom vibrates opposite to its two neighbor-atoms (*Configuration c*). These observations indicate that the trigger of the static mechanical nonreciprocity is a nonreciprocal elasticity.

## Conclusions

The realization of the mechanical nonreciprocity requires breaking either the time-reversal symmetry or the material deformation symmetry. The time-reversal asymmetry was the commonly adopted approach to realize dynamic nonreciprocity. Here, we showed the realization of static nonreciprocity and dynamic nonreciprocity by breaking the material deformation symmetry, which requires nonreciprocal elasticity. We showed that a nonlinear material can break the material deformation symmetry only if it is considered under two different kinematical and kinetical conditions. This makes nonlinear materials (most likely) do not exhibit nonreciprocity, or, if it is fortuitously happened, the achieved nonreciprocity is relatively weak. A strong and effective nonreciprocity requires breaking the material deformation symmetry under the same kinematical and kinetical conditions.

In this study, we investigated the conditions of breaking the material deformation symmetry and the realization nonreciprocity using passive materials. We demonstrated that linear-passive materials can achieve effective static and dynamic nonreciprocities. We demonstrated that achieving nonreciprocity requires a material that can break the deformation symmetry under the same conditions. This material is not essentially required to be nonlinear but nonreciprocal elastic. We developed metamaterials with nonreciprocal linear and nonlinear elasticities. We utilized the developed metamaterials to demonstrate that the trigger of the static nonreciprocity is a nonreciprocal elasticity. We achieved non-trivial topological mechanical systems utilizing nonreciprocal linear and nonlinear elastic materials.

The nonreciprocal elasticity concept developed here will open new venues of the design of metamaterials that can effectively break the material deformation symmetry and achieve, both, static and dynamic nonreciprocity. Approaches that depend on breaking the time-reversal symmetry can only achieve dynamic nonreciprocity, which does not necessary postulate a static nonreciprocity. However, approaches that depend on the attainment of nonreciprocity based on the material deformation asymmetry can realize both static and dynamic nonreciprocity.

## Data Availability

The data that support this study are available within the article.
